# Interventions to Promote Fruit and Vegetable Consumption in Children with Neurodevelopmental Disorders: A Scoping Review

**DOI:** 10.3390/nu17172747

**Published:** 2025-08-25

**Authors:** Laura Torres-Collado, Carolina Ojeda-Belokon, Gema Moreno-Morente, Verónica Company-Devesa, Laura-María Compañ-Gabucio, Manuela García-de-la-Hera

**Affiliations:** 1 Unidad de Epidemiología de la Nutrición, Departamento de Salud Pública, Historia de la Ciencia y Ginecología, Universidad Miguel Hernández (UMH), 03550 Alicante, Spaincojeda@umh.es (C.O.-B.);; 2 Instituto de Investigación Sanitaria y Biomédica de Alicante (ISABIAL), 03010 Alicante, Spain; veronica.company@umh.es; 3 CIBER Epidemiología y Salud Pública (CIBERESP), Instituto de Salud Carlos III, 28029 Madrid, Spain; 4 Departamento de Patología y Cirugía, Universidad Miguel Hernández (UMH), 03550 Alicante, Spain; 5 Being + Doing & Becoming Occupational Research Group (B + D + b), Miguel Hernández University, 03550 Alicante, Spain

**Keywords:** review, neurodevelopmental disorders, children, fruit, vegetable

## Abstract

Background/Objectives: Children with neurodevelopmental disorders (NDDs) frequently present with feeding challenges that can lead to inadequate fruit and vegetable consumption, which can increase their risk of nutritional deficiencies and related health issues. The aim of this scoping review was to describe the main interventions focused on promoting fruit and vegetable consumption in children with NDDs. Methods: Two authors carried out a search strategy in PubMed, Scopus, EMBASE, Web of Science, and PsycINFO using the following terms: “autism OR autistic OR asperger OR rett OR pervasive OR disintegrative OR ‘neurodevelopmental disorder’ OR ‘attention deficit disorder with hyperactivity’”; and the outcome (O): “fruit OR vegetable”. We included all randomized or non-randomized studies, published in English or Spanish, which assessed children’s fruit and vegetable consumption. Results: We included seven articles that applied different interventions, such as a mobile health and behavioral modification program (*n* = 1), repeated food exposure programs (*n* = 2), a play-based program with parental training (*n* = 1); the implementation of Dietary Approaches to Stop Hypertension diet (*n* = 1); an intensive interdisciplinary mealtime behavioral treatment (*n* = 1); and restrictive diets (*n* = 1). Conclusions: Parent-led behavior changes supported by multidisciplinary teams using play, positive reinforcement, and food modification strategies were the main interventions used to increase fruit and vegetable consumption in children with NDDs. This review supports designing evidence-based approaches to improve feeding challenges in this population.

## 1. Introduction

Healthy eating is one of the main determinants of health, as it contributes to the prevention of chronic diseases and premature deaths [1]. In contrast, an unhealthy diet, which is characterized by the consumption of high-calorie foods, sugars, and fats, combined with a reduced intake of foods such as vegetables and fruits [2,3], could be a predictive factor for obesity, as well as other diseases such as diabetes or hypertension [1]. In this sense, 40% to 60% of childhood obesity persists into adulthood [4], possibly because childhood food preferences have been shown to continue into adulthood [5,6]. Therefore, it is crucial to establish healthy habits from childhood to reduce long-term health risks [1,7]. This can be achieved by including foods such as fruits and vegetables, which are rich in minerals, vitamins, fiber, and phytochemicals and have anti-inflammatory and anti-cancer properties [8].

Previous studies showed that children often do not meet the recommended consumption of fruits and vegetables [5], possibly due to their organoleptic characteristics, such as taste or texture [9]. In this sense, several rejection behaviors have been observed, such as being selective with food, known as food selectivity or “picky eating”, which is considered to be a neophobia [10]. This behavior is especially common in children with neurodevelopmental disorders (NDDs) [3]. Numerous studies have described the impact of diet on this population because up to 80% of children with NDDs report chronic dysfunctional eating behaviors, gastrointestinal or sensory problems, respiratory safety risks, or oromotor difficulties that can lead to inadequate nutrition and even increased obesity rates [10,11,12]. In this population, common behaviors include a preference for a restricted variety of foods, difficulty accepting changes, restricted preferences, food-related anxiety, and a tendency to eat in large quantities [3,11,13]. Food selection is often based on taste, texture, temperature, or color [10,14,15], with a preference for soft, processed, less healthy, sweet, and/or salty foods, while protein-rich foods and those that provide vitamins and fiber, such as fruits and vegetables, are avoided [1,3,10,12,13,15,16,17]. These behaviors not only pose a health risk for children with NDDs but also increase parental stress as they need to manage their children’s nutritional intake and the behavioral issues associated with food rejection [3].

Several review studies have explored how dietary patterns and the consumption of specific food groups affect the symptoms of attention-deficit/hyperactivity disorder (ADHD) [18,19], showing that a balanced diet rich in fruits and vegetables reduces behavioral symptoms and improves mental health in this NDD. However, a meta-analysis [14] analyzed whether children with autism spectrum disorder (ASD) consumed fewer fruits and vegetables and showed inconclusive results mainly due to variations in nutritional standards across countries. In a previous publication, we conducted a review focused on dietary assessment tools in children aged 2–12 years with ASD [20]. That article examined the methodologies used to evaluate diet, while the present manuscript addresses a different research question, specifically analyzing interventions designed to increase fruit and vegetable consumption in children with neurodevelopmental disorders. This distinction underlines the novelty of the present work. Although reviews on this topic exist, we have not found any that address our research question: Which dietary interventions aimed at increasing fruit and vegetable consumption in children with NDDs have been studied in intervention studies? Therefore, the objective of this review is to describe the interventions focused on promoting fruit and vegetable consumption in children with NDDs.

## 2. Materials and Methods

A scoping review (SR) was systematically conducted following the guidelines of the PRISMA Extension for Scoping Reviews (PRISMA-ScR) [21] and the methodological standards of the Cochrane Handbook version 6.4 [22]. Detailed methods are provided in Supplementary Materials (Table S1). We chose an SR because it can be used to examine the scope, variety, and characteristics of the evidence on a particular topic [23]. No protocol for this review was published, nor was it registered in PROSPERO or similar. To ensure maximum replicability, we followed the steps below:

### 2.1. Search Strategy

In order to conduct a comprehensive search and to include the maximum number of published studies, we performed a systematic literature search on 15 February 2024. We followed the recommendations of Bramer et al. [24] to carry out our search in four multidisciplinary databases, PubMed, EMBASE, Scopus, and Web of Science, and one psychology-specific database, PsycINFO. We used the same search strategy across all the databases, combining MESH terms using the Boolean operators AND and OR (Table 1). The search terms were grouped into the study population (P): “autism OR autistic OR asperger OR rett OR pervasive OR disintegrative OR ‘neurodevelopmental disorder’ OR ‘attention deficit disorder with hyperactivity’”; and the outcome (O): “fruit OR vegetable”.

Based on previously published reviews, we used NDD diagnoses defined by the Diagnostic and Statistical Manual of Mental Disorders (DSM)-IV instead of those in the current DSM-V manual to avoid excluding potentially relevant articles [25]. In addition, we applied two filters across the different databases: (1) by title and (2) by abstract to refine the search strategy and retrieve studies with clinical relevance [26].

### 2.2. Eligibility Criteria and Study Selection

For inclusion in this review, articles had to be randomized or non-randomized experimental studies that evaluated fruit and vegetable consumption, with full-text availability, published in Spanish or English, and with a study population with a mean age of ≤12 years and ASD or ADHD diagnosis [27].

For the screening process, we used an Excel document in which we compiled all titles previously downloaded from the five databases. After consolidating the data, we removed duplicate titles before beginning the screening process, which consisted of three phases: by title, abstract, and full text. Two authors independently conducted the full screening process (L.T.C. and LM.C.G.), and a third author resolved any discrepancies that arose (M.G.H.).

### 2.3. Data Extraction

Following the guidelines of the Cochrane Handbook [22], we designed and completed three tables. The first “General Characteristics of Included Studies”, presents the following study characteristics: author/year, study design, sample, country, participants, diagnosis, intervention/comparison, evaluation, and outcome variables. The second “Summary of Findings of the included studies”, provides information directly related to our research question, focusing on aspects of fruit and vegetable consumption: author/year, participants and diagnosis, dietary variable studied and measurement instruments, categorization of analyses, and main results. The third table “Risk of bias of the included studies”, contains indicators related to the risk of bias in the included articles: author/year, main limitations, funding, and conflicts of interest.

### 2.4. Quality Assessment

We have not carried out a quality assessment. It is not mandatory to do so because the primary objective of an SR is to expand knowledge on a particular topic and not to evaluate it [28]. However, we included a table summarizing the limitations of the included studies to facilitate critical reading.

## 3. Results

**Figure 1:**
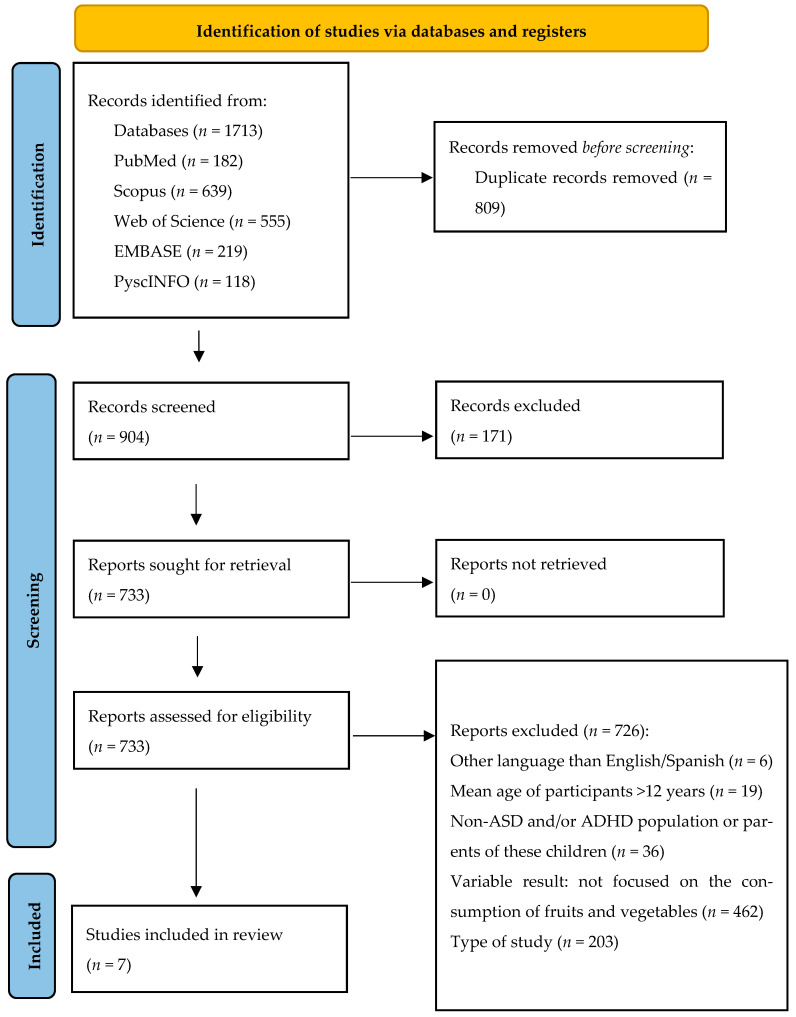
Flowchart of the study selection process.

A total of 1713 articles were identified across all databases consulted. Duplicates were removed (*n* = 809), leaving 904 articles for screening by title and abstract. Of these, 733 articles were selected for full-text screening. The seven that met the inclusion criteria were included in this scoping review (Figure 1).

### 3.1. Main Characteristics of the Included Studies

Table 2 summarizes the main characteristics of the included studies. This review includes four randomized clinical trials [29,30,31,32] and three non-randomized clinical trials [33,34,35]. Of these, three were conducted in Asia (South Korea [31], Iran [32], and China [35]), two in Europe (Poland [34] and Iceland [29]), and two in the United States [30,33]. Two articles were published in 2023 [30,34], two in 2021 [29,32], two in 2020 [33,35], and one in 2018 [31].

### 3.2. Population in the Included Studies

Included studies had sample sizes of 40 [30,31], 50 [33,35], 80 [32,34], and 190 [29] participants. In three studies, the intervention population consisted only of children [31,32,35], while in the other four studies, parents were also included [29,30,33,34].

Although the average age of participants was 7 years, some studies focused on younger children with a mean age between 3 and 7 years [31,33,34], while others included children aged 6 to 15 years [29,30,32,35]. Most of the children included in these studies had an ASD diagnosis [29,30,31,33,34,35], whereas two studies included children diagnosed with ADHD [29,32]. To evaluate these diagnoses, three studies used the DSM IV/V Edition [30,32,35], two studies used information provided by parents [31,33], one used standardized diagnostic instruments and protocols [29], and one used the International Statistical Classification of Diseases and Related Health Problems 10th Revision [34].

Recruitment in the included studies was carried out in one study through media outreach [29], while in the others, it was carried out in healthcare centers or services that the participants were already attending [30,31,32,33,34,35].

### 3.3. Interventions Conducted in Included Studies

Six studies included both intervention and control groups [29,30,31,32,33,34], two of which had control groups composed of children without any NDD diagnosis [29,33]. Only one study did not include a control group [35]. Interventions and comparators were as follows: a nutritional intervention using mobile health technology and behavioral modification strategies versus a brochure with nutritional recommendations [30], a series of 24 activities based on repeated exposure to vegetables versus usual treatments of control participants [31], a play-based program, “Taste Education”, consisting of parental training and kitchen-based intervention versus the same intervention at a later point [29], an implementation of a Dietary Approaches to Stop Hypertension (DASH) diet based on fruits, vegetables, seeds, legumes, and dairy products versus a control diet similar to their usual diet [32], an intensive interdisciplinary mealtime behavioral treatment together with a varied diet in children with NDDs versus a control group without diagnosis of NDDs [33], an implementation of casein- and/or gluten-free restrictive diets versus a control group with a regular diet [34], and finally, a four-week fruit and vegetable exposure program without a control group [35]. The outcomes of these interventions are summarized in Table 3.

### 3.4. Study Variables: Fruit and Vegetable Consumption

The variables analyzed across the studies focused, on the one hand, on promoting the consumption of those foods, which are in line with a healthy diet, such as fruits and vegetables [29,30,31,33,34,35] and legumes and seeds [29,32,34]. On the other hand, they focused on reducing the consumption of unhealthy foods, such as those rich in fats and sugars, or sugar-sweetened beverages [30]. In another study, fruit and vegetable consumption was used to alleviate ADHD symptoms [32].

Five of the included studies focused on the consumption of both fruits and vegetables [29,30,32,33,35], while two focused exclusively on vegetables [31,34], and only one study specifically reported the type of vegetable used [34]. In addition, to evaluate changes in fruit and vegetable consumption, five studies used scales [29,30,33,34,35], three 24 h dietary records [30,31,32], two food checklists [29,33], and only two evaluated the number of foods consumed [31,35].

### 3.5. Assessment Tools

The most commonly used assessment tools to evaluate changes in fruit and vegetable consumption were 24 h dietary records, completed on three different days of the week (*n* = 3) [30,31,32], which documented everything the child had eaten the previous day and evaluated the presence or absence of the target foods. In addition, two studies used the *Brief Assessment of Mealtime Behavior* (BAMBI) and the *Brief Assessment of Mealtime Behavior in Children* (BAMBIC), both of which assessed mealtime behaviors commonly observed in children with ASD, which can influence their eating habits [33,35]. One of these studies also used a food inventory in which caregivers indicated whether the child consumed specific foods [33], as well as the *About Your Child’s Eating Scale* (AYCE) to assess mealtime behaviors [33]. Another study used the *Food Frequency Questionnaire* (FFQ) to evaluate the frequency of consumption across 62 food groups [34], while The *Children’s Eating Behaviour Questionnaire* (CEBQ) was used to assess food refusal [29], and the *Child Feeding Questionnaire* (CFQ) was used to measure food selectivity [30] (Table 3).

### 3.6. Main Limitations Reported by the Included Studies

Table 4 outlines the primary limitations identified by the authors of the included studies. Among these, the most frequently mentioned was small sample size (*n* = 4) [30,31,33,35], followed by participant variability (*n* = 3) [29,31,33] and difficulties encountered during measurement processes (*n* = 3) [29,32,33]. Three studies highlighted difficulty when comparing interventions due to the absence of a control group [29,33,35], while two mentioned a high dropout rate among participants and short study duration [30,33]. In addition, specific limitations were reported, which varied across studies.

## 4. Discussion

The most frequently identified interventions to promote fruit and vegetable consumption in children with NDDs relied on behavior modification strategies, which were delivered through a multidisciplinary approach, with active parental involvement playing a central role.

The majority of the studies included in this review were conducted in Asian countries. A previous study by Kang et al. [36] suggested that there is a growing interest in Asian countries, as most previous studies have been carried out in Western countries. In this sense, Yan et al. [37] point to the steadily rising prevalence of ADHD over the past decades in Asian children and the necessity of studying them. Moreover, Ismail et al. [38] reported insufficient nutritional knowledge among caregivers of children with ASD in Malaysia. The authors highlighted the need to incorporate a specific nutrition module for children with ASD in national dietary guidelines, given the increasing prevalence of ASD diagnoses. Thus, the increasing number of studies in this population reflects this interest and allows us to explore cultural differences that may influence feeding behaviors and challenges among children with NDDs. Recognizing these cultural distinctions, such as differences in caregiver beliefs and parenting styles, is essential in order to adapt and enhance the effectiveness of feeding interventions originally developed in Western contexts.

It is important to highlight the lack of recent studies from Mediterranean countries included in this SR. Populations in these countries have traditionally followed the Mediterranean diet, which is characterized by a high intake of fruits, vegetables, legumes, and healthy fats. One possible explanation for the lack of research could be the assumption that adherence to this dietary pattern reduces the need for dietary intervention trials [39]. However, this assumption is particularly concerning, given evidence that indicates that Spanish children with ASD often show significantly lower adherence to the Mediterranean diet and higher levels of inflammatory biomarkers compared to their TD peers [40]. In addition, several studies have reported that low adherence to the Mediterranean diet in children is associated with an increased risk of an ADHD diagnosis [39,41]. To sum up, while Asian countries appear to prioritize nutritional intervention-based research, Mediterranean countries’ presumption of dietary adequacy may contribute to the underrepresentation of fruit- and vegetable-based interventions for Mediterranean children with NDDs.

An additional relevant aspect reflected in these studies is that the majority of participants were boys who had been diagnosed with ASD and ADHD, and only one study explicitly acknowledges this as a concern [32]. One possible explanation for this disparity is the underdiagnosis or misidentification of girls with NDDs, due to differences in symptom type and severity. This may contribute to differences in prevalence ratios of diagnoses between boys and girls, which may result in reduced access of girls to intervention resources and lower representation in research studies [42,43]. Nevertheless, sex differences in the prevalence of feeding-related difficulties persist. Although these issues are more commonly reported in boys, several studies suggest that feeding difficulties may be even more prevalent in girls, a trend that seems to vary depending on the country where the study is conducted [44].

It is also important to mention that a considerable number of studies involving children with ADHD use diet solely as a means of reducing core symptoms of the disorder instead of focusing on the improvement of diet quality and food variety. This could be considered a lost opportunity as these types of studies can be a preliminary step toward promoting adherence to a balanced diet within this population. In fact, several studies included in systematic reviews and meta-analyses agree that although dietary interventions do not significantly reduce hyperactivity, they do contribute to decreasing inattention, aggression, and emotional dysregulation and may serve as a protective factor against ADHD [18,32,38,45,46].

In addition, it is important to mention elimination diets, such as the one employed in the study by Jessa et al. [34], which increased the intake of vegetables, seeds, nuts, and berries, thereby enhancing children’s predisposition toward these foods compared to a group that maintained their usual diet. However, such diets should not be prescribed unless there is clear evidence of a diagnosed food intolerance or gastrointestinal disorder, as noted in several studies [19,47,48].

Among the included studies in this SR, two [32,34] focused mainly on dietary modification, while the others [29,30,31,33,35] used different strategies. Interventions aimed at increasing fruit and vegetable intake in children with and without NDDs were primarily based on three strategies: behavioral modification, involvement of parents as active agents, and multidisciplinary treatment approaches, a result which was supported by a meta-analysis conducted by Marshall et al. [49]. Similarly, in the study by Ausderau et al. [50], they determined that the most frequent therapy strategies used by parents were environmental and food preparation modifications, positive reinforcement, and the use of play and motivation as tools to elicit behavioral change in their children. Moreover, studies by Laud et al. [51] and Johnson et al. [52] showed that interventions based on behavioral modification, particularly those implemented by parents, effectively increase food consumption and reduce stress levels. In line with this result, we found that interventions that combined these characteristics had the greatest impact, increasing mealtime enjoyment for up to six months post-intervention [29,30,35].

The articles included in this SR used different tools to assess food refusal, children’s behavior during meals, and frequency of consumption. Of these evaluation tools, the most commonly used were the BAMBI scale and the 24 h dietary record. These findings align with those reported in an SR [20] that highlighted that the use of the BAMBI scale and the 24 h dietary record provides valuable insights into children’s eating behaviors due to their validity, ease of completion by parents, and the additional information they offer regarding the family environment and perceptions.

The difference between the fruit and vegetable consumption of children with and without NDDs remains a controversial topic. While numerous studies reported lower intake among children with NDDs, a previous meta-analysis [14] found that these children not only consumed more fruits and vegetables than their TD peers but even reached the recommended consumption levels. In our SR, Seiverling et al. [33] reported no significant differences in fruit consumption between the NDD and non-NDD groups. Similarly, Thorsteinsdottir et al. [29] indicated that changes in fruit and vegetable consumption post-intervention were not significant between the two groups, while Canals-Sans et al. [53] observed similar consumption levels in both groups. These findings may be due to differences in diagnostic characteristics. Children generally prefer high-calorie, low-fiber diets, which do not include legumes, nuts, fruits, and vegetables, but in children with NDDs, this pattern tends to be intensified due to their behavioral rigidity, stress, and their families’ difficulties in managing this situation. In addition, one of the main differences observed in intervention outcomes was that children with NDDs needed more time to internalize and generalize changes across different contexts than children without NDDs [29,33].

This SR has several limitations. Our findings may have been influenced by selection bias, which is a common limitation in review studies. This bias may have been increased by the use of title and abstract search filters and the selection of only those articles published in English or Spanish, which may have led to potentially overlooking relevant studies published in other languages. However, it is important to note that English is the universal language of science, and most research is published in this language. Moreover, we could consider the exclusive inclusion of clinical trials as a limitation because some had small sample sizes, which can represent a source of bias. Nevertheless, it should be emphasized that the included studies consist of both randomized and non-randomized clinical trials, which are among the study designs that provide the highest level of scientific evidence in intervention research. This strengthens the reliability of the conclusions despite the modest sample sizes. In addition, changes in the classification criteria for ASD could constitute a limitation, as some outdated diagnostic terms were included in the search strategy to avoid excluding potentially relevant studies. Furthermore, the exclusion of children with low-functioning ASD complicates the generalization of the results across the entire autism spectrum, as communication difficulties, both in expression and comprehension, often hinder participation in proposed interventions. In addition, we have not taken into account cultural differences in dietary practices between the countries where the included studies were conducted. Lastly, the quality of the included articles was not formally assessed. However, to enhance the transparency of the review, a table summarizing the main limitations and conflicts of interest disclosed by the original authors has been provided.

This review also presents some strengths. It synthesizes the most recent evidence because the included studies were published within the past seven years. Our review provides a robust overview of the differences in the consumption of fruits and vegetables in children with and without NDDs. Moreover, several knowledge gaps were identified: (1) there is a limited number of published studies in Europe, particularly from countries with a Mediterranean diet traditionally rich in fruit, vegetables, and fresh products; (2) no clear intervention strategy has yet been established for the management of restricted diets in children with NDDs; and (3) there is insufficient research addressing the influence of sex and country of origin on the prevalence of feeding difficulties.

The findings of this review have practical implications for both clinical practice and future research. Clinically, healthcare professionals working with children with NDDs should consider structured interventions to increase fruit and vegetable intake, involving caregivers, schools, and therapists to tailor strategies according to each child’s preferences and sensory sensitivities. For future trials, larger, high-quality studies are needed to evaluate the effectiveness of different intervention strategies, their long-term adherence, and potential impact on overall diet quality. Additionally, culturally adapted interventions should be developed to ensure that programs are applicable in diverse settings. These recommendations may guide both clinical practice and the design of future research, supporting the development of effective, evidence-based dietary interventions for children with NDDs.

## 5. Conclusions

The most effective interventions for increasing fruit and vegetable consumption in children with NDDs are those in which parents, as primary caregivers, modify their children’s behavior under the guidance of a multidisciplinary team of healthcare professionals. The most commonly used intervention strategies include the use of play, positive reinforcement, and food modification. Our overview could help multidisciplinary teams to design interventions for addressing daily feeding challenges in children with NDDs and ensure that such approaches are grounded in updated evidence.

## Figures and Tables

**Table 1:** Databases and search strategies used.

Databases	Search Strategy 15 February 2024	Results
PubMed		
#1	“autism s” [All Fields] OR “autisms” [All Fields] OR “autistic disorder” [MeSH Terms] OR (“autistic” [All Fields] AND “disorder” [All Fields]) OR “autistic disorder” [All Fields] OR “autism” [All Fields] OR (“autistic disorder” [MeSH Terms] OR (“autistic” [All Fields] AND “disorder” [All Fields]) OR “autistic disorder” [All Fields] OR “autistic” [All Fields] OR “autistics” [All Fields] OR “autists” [All Fields]) OR (“asperger” [All Fields] OR “asperger s” [All Fields] OR “aspergers” [All Fields]) OR “rett” [All Fields] OR (“pervasive” [All Fields] OR “pervasively” [All Fields] OR “pervasiveness” [All Fields]) OR “disintegrative” [All Fields] OR “neurodevelopmental disorder” [All Fields] OR “attention deficit disorder with hyperactivity” [All Fields]	138,904
#2	“fruit” [MeSH Terms] OR “fruit” [All Fields] OR “fruits” [All Fields] OR “fruit s” [All Fields] OR “fruited” [All Fields] OR “fruiting” [All Fields] OR “vegetables” [MeSH Terms] OR “vegetables” [All Fields] OR “vegetable” [All Fields]	293,154
	#1 AND #2	481
	#1 AND #2 Title and Abstract	182
Scopus		
#1	ALL ((autism OR autistic OR asperger OR rett OR pervasive OR disintegrative OR “neurodevelopmental disorder” OR “attention deficit disorder with hyperactivity”))	871,827
#2	ALL ((fruit OR vegetable))	1,868,552
	#1 AND #2	15,078
	#1 AND #2 Title and Abstract	639
EMBASE		
#1	‘autism’/exp OR autism OR autistic OR asperger OR rett OR pervasive OR disintegrative OR ‘neurodevelopmental disorder’/exp OR ‘neurodevelopmental disorder’ OR ‘attention deficit disorder with hyperactivity’/exp OR ‘attention deficit disorder with hyperactivity’	3,021,033
#2	fruit OR vegetable	254,745
	#1 AND #2	9670
	#1 AND #2 Title and Abstract	219
Web of Science		
#1	(autism OR autistic OR asperger OR rett OR pervasive OR disintegrative OR “neurodevelopmental disorder” OR “attention deficit disorder with hyperactivity”) (Topic)	396,720
#2	(fruit OR vegetable) (Topic)	1,180,083
	#1 AND #2	1181
	#1 AND #2 Title and Abstract	555
PsycINFO		
#1	(autism OR autistic OR asperger OR rett OR pervasive OR disintegrative OR “neurodevelopmental disorder” OR “attention deficit disorder with hyperactivity”)	135,746
#2	(fruit OR vegetable)	12,855
	#1 AND #2	163
	#1 AND #2 Title and Abstract	118

**Table 2:** General characteristics of included studies.

Author, Year	Design	Sample (*n*), Country	Participants	Diagnosis	Intervention/Comparator	Evaluation	Outcome Variables
Kim et al. [31], 2018	RCT	42, South Korea Loss to follow-up (*n* = 15)	27 children with ASD. Age between 3.13 and 5.34 years (mean age 4.23)	ASD with a moderate degree of food selectivity	6-month repeated exposure program/usual treatments of control participants	Pre- and post-evaluation	Vegetable consumption
Seiverling et al. [33], 2020	nRCT	52, United States Loss to follow-up (*n* = 0)	52 children with ASD and caregivers. Age range NS (mean age 4.38)	ASD, other special needs, and no special needs	2 years of intensive interdisciplinary behavioral treatment/control group without diagnosis of NDDs	Pre- and post-evaluation	Eating behavior, diet variety, and the family mealtime environment
Chung et al. [35], 2020	nRCT	56, China Loss to follow-up (*n* = 0)	56 children with ASD. Age between 8 and 15 (mean age 10.7)	ASD	4-week program of fruit and vegetable exposure, three times per week/NA	Pre- and post-evaluation	Approval of fruits and vegetables by food transformation
Khoshbakht et al. [32], 2021	RCT	86, Iran Loss to follow-up (*n* = 6)	80 children with ASD. Age between 6 and 12 years (mean age NS)	ADHD	12-week program of DASH diet/control diet	Pre-, post- and every month evaluation	Hyperactivity and impulsivity, emotional symptoms, conduct problems, peer relationship problems, prosocial behavior
Thorsteinsdottir et al. [29], 2021	RCT	190, Iceland Loss to follow-up (*n* = 109)	81 children with ASD and ADHD and caregivers. Age between 8 and 12 years (mean age 10.4)	Fussy eaters with and without NDDs	7-week Taste Education intervention/matched, delayed intervention	Pre-, post-, and 6-month follow-up evaluation	Changes in fussy eating
Jessa et al. [34], 2023	nRCT	88, Poland Loss to follow-up (*n* = 0)	88 children with ASD. Age between 3 and 7 years (mean age 4.04)	ASD	On year of a gluten-free diet, a gluten- and casein-free diet, or a regular diet	Pre- and post-evaluation	Consumption of nuts, seeds, berries, and cruciferous vegetables
Kral et al. [30], 2023	RCT	46, United States Loss to follow-up (*n* = 8)	38 children with ASD and caregivers. Age between 6 and 10 years (mean age 8.65).	Picky eaters, children with ASD	3-month trial of a mobile health nutrition intervention/education group	Pre- and post-evaluation	Consumption of fruits and vegetables, salty and sugary snacks, and sugar-sweetened beverages

ADHD: attention-deficit/hyperactive disorder; ASD: autism spectrum disorder; NA: not applicable; NDD: neurodevelopmental disorder (ASD or/and ADHD); NS: not stated; RCT: randomized controlled trial; nRCT: non-randomized controlled trial.

**Table 3:** Summary of findings of the included studies.

Author, Year	Participants and Diagnosis	Dietary Variable Studied and Measurement Instruments	Categorization of Analyses	Main Results
Kim et al. [31], 2018	27, ASD	Touch, taste, and consumption of the vegetables selected during the activities. Nutritional intake before and after the intervention.	During the activities, touching and tasting of the selected vegetables were coded on an interval scale (0–12), while consumption was recorded based on the number of pieces consumed (0–60). Following the activities, nutritional intake was assessed using the 24 h dietary recall method over three self-selected days.	An increase in vegetable consumption was observed in the exposure group within the experimental setting. No significant group differences were found in overall nutritional intake.
Seiverling et al. [33], 2020	52, NDDs and not NDDs	Mealtime behavior and environment and variety of foods included in the diet (AYCE, BAMBIC, 70-item food inventory).	Resistance to eating, a positive mealtime atmosphere, caregiver aversion, food refusal, limited dietary variety, and disruptive behaviors during meals. Dietary variety included 20 types of fruits, 23 types of vegetables, 12 sources of protein, eight types of grains, and seven dairy products.	Outcomes related to consumption improved, except for fruit intake among children diagnosed with ASD, compared to children without ASD or with other NDDs.
Chung et al. [35], 2020	56, ASD	Fruit and vegetable acceptance during the intervention, habitual fruit and vegetable consumption, and mealtime behavior.	Food acceptance was assessed by calculating the difference in the weight of food samples before and after consumption. Intake of fruits and vegetables was evaluated using questionnaires, while mealtime behavior was measured using the BAMBI questionnaire.	Fruit consumption frequency increased, especially for bananas, but vegetable consumption did not.
Khoshbakht et al. [32], 2021	80, ADHD	AHDH severity (ACS, SNAP-IV, and SDQ).	Behavioral questions focus on hyperactivity and inattention, impulsivity, emotional symptoms, conduct problems, peer relationship problems, and prosocial behavior.	Adherence to the DASH diet improved ADHD symptoms compared to the control diet.
Thorsteinsdottir et al. [29], 2021	81, ASD, ADHD, and NDDs	Children’s fussy eating was assessed using the CEBQ, while food intake and acceptance were measured through a parent-report questionnaire.	The CEBQ measured the following dimensions: food fussiness, slowness in eating, food responsiveness, emotional over-eating, emotional under-eating, enjoyment of food, desire to drink, and satiety responsiveness. Food intake was categorized into three indices: fruits, nuts, and seeds; vegetables; and a total food intake index.	Acceptance of vegetables, nuts, and seeds increased in both groups. The intervention also led to a reduction in food fussiness and an increase in enjoyment of food.
Jessa et al. [34], 2023	88, ASD	Consumption of nuts, seeds, berries, and cruciferous vegetables assessed through an FFQ.	FFQ including 62 different food groups.	Children who maintained a casein-free and gluten-free diet increased their consumption of vegetables, seeds, nuts, and berries.
Kral et al. [30], 2023	38, ASD	Food intake using the telephone-based 24 h dietary recall method.	Individual items coded to salty and sugary snacks, sugar-sweetened beverages, and fruits and vegetables.	The mobile health nutrition group increased their fruit and vegetable intake compared with the education group. The education group decreased calories consumed from savory snacks, while the mobile health nutrition group increase these calories.

ACS: 10-item Conner’s scale; ADHD: attention-deficit/hyperactivity disorder; ASD: autism spectrum disorder; AYCE: About Your Child’s Eating scale; BAMBI: Brief Autism Mealtime Behavior Inventory; BAMBIC: Brief Assessment of Mealtime Behavior in Children; CEBQ: Children’s Eating Behavior Questionnaire; DASH: Dietary Approaches to Stop Hypertension; FFQ: Food Frequency Questionnaire; NDD: neurodevelopmental disorder; SDQ: Strength and Difficulties Questionnaire; SNAP-IV: 18-item Swanson, Nolan, and Pelham.

**Table 4:** Risk of bias of the included studies.

Author, Year	Main Limitations	Funding	Conflict of Interest
Kim et al. [31], 2018	Small sample size. Recruitment method. Variability in participant characteristics. Lack of significant nutritional changes.	Not stated.	None declared.
Seiverling et al. [33], 2020	Small sample size. Variety of diagnoses. Comorbidities among diagnoses. Varying study duration. Dropout of 13 children. Difficulties with assessment measures.	Not stated.	None declared.
Chung et al. [35], 2020	Small sample size. Only three fruits and three vegetables were used. Food modification methods cannot be generalized to all foods. No control group included.	Not stated.	None declared.
Khoshbakht et al. [32], 2021	Only a 3-day food record was used to assess adherence. There was only one girl with ADHD. The diets did not differ much from each other. No food was provided to the participants.	Not stated.	None declared.
Thorsteinsdottir et al. [29], 2021	There was no comparison between the parental education groups. The amount of food consumed by each child was not weighed. Changes in medication dosages were not recorded. Non-functional children with ASD were not included. Results were based on parents’ perceptions rather than behavioral observations. Many parents worked full-time and a few lived in single-parent households.	Not stated.	None declared.
Jessa et al. [34], 2023	Not declared.	Not stated.	None declared.
Kral et al. [30], 2023	Small sample size. High dropout rate. Greater variety of food examples in the intervention group compared to the control group. Intensity and duration of the study.	The Eunice Kennedy Shriver National Institute of Child Health and Human Development supported the research.	Financial conflicts of interest related to the intellectual property of the mHealth nutrition intervention tested in this clinical trial.

ADHD: attention-deficit/hyperactivity disorder; ASD: autism spectrum disorder; mHealth: mobile health.

## Data Availability

The original contributions presented in this study are included in the article/Supplementary Materials. Further inquiries can be directed to the corresponding author.

## Supplementary Materials

The following supporting information can be downloaded at https://www.mdpi.com/article/10.3390/nu17172747/s1, Table S1: PRISMA-ScR Checklist.

## Abbreviations

The following abbreviations are used in this manuscript:

NDDs	Neurodevelopmental disorder
ASD	Autism Spectrum Disorder
ADHD	Attention-deficit/hyperactivity disorder
PRISMA-ScR	PRISMA extension for Scoping Reviews
